# 

**Published:** 1974-01

**Authors:** 


					M
mm
lSSs=@6^
-A. v V
^1
NATIONAL LIBRARY OF MEWCtNi
INDEX COPY
RECD. OCT 1 W/b
index NOV 10
input ^ - ~u"
S-fc-C fS!
A&IlIo- .3
I X GATE
:Jsjn
21
i- -*
;;j INDEX MEDICUS
/? ? i hi ?    ."i1:
.
JANUARY, 1974
VOL. 89 (I) No. 329

				

